# Supplementary material for: Shared Treg dysregulation underlies psoriasis vulgaris and atopic dermatitis through the PHF19-PRC2 epigenetic axis
Source: Front Immunol. 2026 Aug 20;17:1932788. doi: 10.3389/fimmu.2026.1932788 (PMC13538097; doi:10.3389/fimmu.2026.1932788)
Supplement: Supplementary file 3 [file DataSheet3.zip › Additional files/Additional file 6/60.RTKN2.forest.pdf]

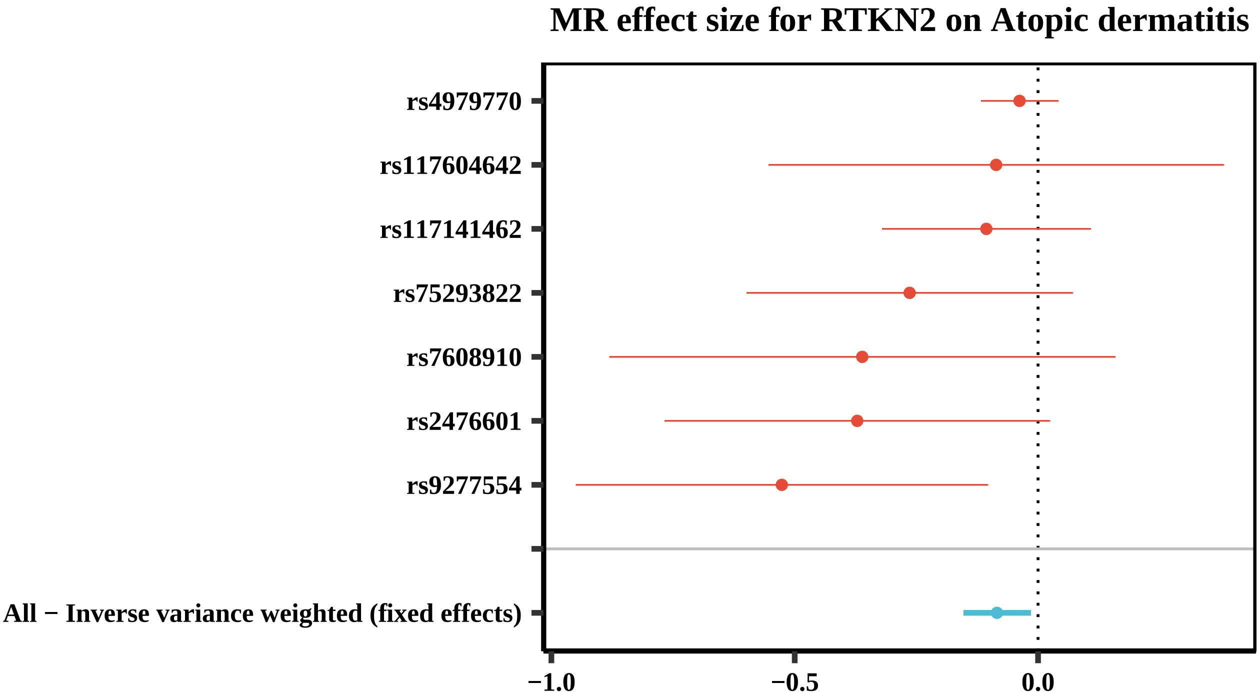
MR effect size for RTKN2 on Atopic dermatitis
rs4979770
rs117604642
rs117141462
rs75293822
rs7608910
rs2476601
rs9277554
All − Inverse variance weighted (fixed effects)
−1.0
−0.5
0.0
